# Predictive Value of Multiparametric MRI for Response to Single-Cycle Induction Chemo-Immunotherapy in Locally Advanced Head and Neck Squamous Cell Carcinoma

**DOI:** 10.3389/fonc.2021.734872

**Published:** 2021-10-21

**Authors:** Konstantin Hellwig, Stephan Ellmann, Markus Eckstein, Marco Wiesmueller, Sandra Rutzner, Sabine Semrau, Benjamin Frey, Udo S. Gaipl, Antoniu Oreste Gostian, Arndt Hartmann, Heinrich Iro, Rainer Fietkau, Michael Uder, Markus Hecht, Tobias Bäuerle

**Affiliations:** ^1^ Institute of Radiology, University Hospital Erlangen, Erlangen, Germany; ^2^ Institute of Pathology, University Hospital Erlangen, Erlangen, Germany; ^3^ Department of Radiation Oncology, University Hospital Erlangen, Friedrich-Alexander-Universität Erlangen-Nürnberg, Erlangen, Germany; ^4^ Comprehensive Cancer Center Erlangen-European Metropolitan Region of Nuremberg (CCC ER-EMN), Erlangen, Germany; ^5^ Department of Otolaryngology - Head & Neck Surgery, University Hospital Erlangen, Erlangen, Germany

**Keywords:** magnetic resonance imaging, dynamic contrast-enhanced imaging, DCE-MRI, head and neck cancer, immunotherapy, multiparametric MRI

## Abstract

**Objectives:**

To assess the predictive value of multiparametric MRI for treatment response evaluation of induction chemo-immunotherapy in locally advanced head and neck squamous cell carcinoma.

**Methods:**

Twenty-two patients with locally advanced, histologically confirmed head and neck squamous cell carcinoma who were enrolled in the prospective multicenter phase II CheckRad-CD8 trial were included in the current analysis. In this unplanned secondary single-center analysis, all patients who received contrast-enhanced MRI at baseline and in week 4 after single-cycle induction therapy with cisplatin/docetaxel combined with the immune checkpoint inhibitors tremelimumab and durvalumab were included. In week 4, endoscopy with representative re-biopsy was performed to assess tumor response. All lesions were segmented in the baseline and restaging multiparametric MRI, including the primary tumor and lymph node metastases. The volume of interest of the respective lesions was volumetrically measured, and time-resolved mean intensities of the golden-angle radial sparse parallel-volume-interpolated gradient-echo perfusion (GRASP-VIBE) sequence were extracted. Additional quantitative parameters including the T1 ratio, short-TI inversion recovery ratio, apparent diffusion coefficient, and dynamic contrast-enhanced (DCE) values were measured. A model based on parallel random forests incorporating the MRI parameters from the baseline MRI was used to predict tumor response to therapy. Receiver operating characteristic (ROC) curves were used to evaluate the prognostic performance.

**Results:**

Fifteen patients (68.2%) showed pathologic complete response in the re-biopsy, while seven patients had a residual tumor (31.8%). In all patients, the MRI-based primary tumor volume was significantly lower after treatment. The baseline DCE parameters of time to peak and wash-out were significantly different between the pathologic complete response group and the residual tumor group (p < 0.05). The developed model, based on parallel random forests and DCE parameters, was able to predict therapy response with a sensitivity of 78.7% (95% CI 71.24–84.93) and a specificity of 78.6% (95% CI 67.13–87.48). The model had an area under the ROC curve of 0.866 (95% CI 0.819–0.914).

**Conclusions:**

DCE parameters indicated treatment response at follow-up, and a random forest machine learning algorithm based on DCE parameters was able to predict treatment response to induction chemo-immunotherapy.

## Introduction

Cancer immunotherapy is an emerging and highly promising therapeutic approach in oncology. Immune checkpoint inhibitors (ICIs), such as inhibitors targeting PD-1, PD-L1, and CTLA-4, are approved for the treatment of different cancer types such as melanoma, lung cancer, and head and neck squamous cell carcinoma (1–8). Although effective, the overall response rates for ICIs are approximately 15 to 20% for advanced melanoma and non-small-cell lung cancer, while in other tumor types the clinical response varies even more—from approximately 10% to greater than 50% of patients (9–11). Therefore, patient selection is crucial. An accurate prediction of response prior to potential ICI treatment is desirable, but this is still not established. Predictive biomarkers such as tumor PD-L1 expression, microsatellite instability status, and tumor mutational burden show an association with clinical response among different cancer types (12–17). These predictive markers require invasive tumor biopsy and the results may not be representative because of tumor heterogeneity (18). Attempts to correlate genetic heterogeneity in biopsy samples with FDG-SUV or ADC values in PET/MRI were not successful in a small cohort of patients with head and neck cancer (19). More recent approaches focus on the predictive value of peripheral blood immune cells (20).

Another challenge with ICIs is therapeutic evaluation and follow-up. Immunotherapy can cause hyperprogression, an acceleration of tumor growth after treatment, and pseudoprogression, an initial increase in tumor size followed by morphological regression; furthermore, a mixed response with shrinkage and growth of lesions has been reported (21–23). Initial morphological imaging alone may be misleading; therefore, revised Response Evaluation Criteria in Solid Tumors (RECIST) such as iRECIST and imRECIST have been established (23, 24). These criteria facilitate and standardize follow-up, but in many cases uncertainty remains. Functional imaging, e.g. ^18^F-fluorodeoxyglucose (^18^F-FDG) PET/CT, is commonly used to evaluate the tumor response after therapy using the standardized uptake value (SUV) and total lesion glycolysis (TLG). Response evaluation is performed by measuring the SUV normalized by lean body mass (SUL); an increase in SUL peak > 30% or the appearance of a new lesions is considered progressive disease (25). Although ^18^F-FDG PET/CT can provide additional information after treatment with chemotherapeutics, ^18^F-FDG accumulates in inflamed tissue and can lead to false-positive results due to pseudoprogression (26).

Dynamic contrast-enhanced (DCE) MRI is an important diagnostic tool for several tumor types, e.g. prostate and breast cancer. DCE MRI in breast cancer helps to screen high-risk patients and is used to monitor the response to therapy and detect carcinoma *in situ* (27, 28). In multiparametric MRI, the apparent diffusion coefficient (ADC), tumor volume and the difference of the DCE parameter Ktrans before and after chemoradiation are correlated with the pathologic response post chemotherapy for rectal cancer (29, 30). For head and neck cancer, DCE MRI facilitates the diagnosis of cervical lymph node metastases (31). Given the cost of treatment with ICIs, the fact that only a subset of patients responds to this therapy, and the difficulties in response evaluation, a noninvasive predictor of therapy response is essential for personalized cancer immunotherapy. Most of the studies evaluating radiomic biomarkers in head and neck squamous cell carcinoma (HNSCC) focus on CT; few used MRI or PET/CT, and a validation cohort was only used in slightly more than half of the studies (32).

This study aimed to create a predictive model for the response assessment of induction chemo-immunotherapy using multiparametric MRI in advanced HNSCC with biological validation through biopsy.

## Materials and Methods

### Study Design, Data Source, and Image Acquisition

In this unplanned secondary single-center analysis of the CheckRad-CD8 trial, the predictive value of multiparametric MRI was studied. Patients with locally advanced, histologically confirmed HNSCC stage III–IVb (according to TNM classification of malignant tumors 8^th^ edition) of the oral cavity, oropharynx, hypopharynx, or supraglottic larynx received contrast-enhanced MRI at baseline and in week 4 after induction therapy with cisplatin [30 mg/m² body surface area (BSA)] on days 1–3 and docetaxel (75 mg/m² BSA) on day 1. The anti-CTLA4 ICI tremelimumab (75 mg fixed dose) and the anti-PDL1 durvalumab (1500 mg fixed dose) were administered on day 5. In week 4, endoscopy with representative re-biopsy was performed to assess pathologic response and the density of intratumoral CD8+ cells. In case no residual tumor was clinically detected, biopsies were taken from the primary tumor region. Patients with biopsies with no remaining tumor in a sufficiently covered tumor bed in the re-biopsy were scored as pathologic complete response (pCR). The results from the first interim analysis of the induction period of the CheckRad-CD8 trial on safety and efficacy were recently reported by Hecht et al. (33).

All MRI examinations were performed on a 3 Tesla MRI following the institutional reference protocol (Magnetom Vida; Siemens Healthineers, Erlangen, Germany).

- pre-contrast short-tau inversion recovery (STIR) T2-weighted (T2w) slice thickness = 4 mm, coronal
- pre-contrast T2w STIR, slice thickness = 3 mm, transversal
- pre-contrast T1-weighted (T1w) turbo spin echo (TSE), slice thickness = 3 mm, transversal
- diffusion-weighted imaging with apparent diffusion coefficient (ADC) map, slice thickness = 5 mm, transversal
- gadobutrol (Gadovist, Bayer HealthCare Pharmaceuticals) was administered to the patients (0.1 mmol/kg), followed by a 30-mL saline flush *via* a power injector at a rate of 1 mL/s
- Golden-angle radial sparse parallel (GRASP) technique (Siemens Healthineers) was applied to a transversal T1-weighted volume-interpolated gradient-echo perfusion sequence (VIBE), started 8 seconds before contrast injection, obtained for each patient with the minimum temporal offset available (2.5 s) over 338 s of total acquisition time (34).
- post-contrast T1w TSE with fat saturation (fs), slice thickness = 3 mm, transversal
- post-contrast T1w TSE fs-dixon, slice thickness = 3 mm, coronal

A total of 22 patients from October 2019 to October 2020 were included. In one case, DCE imaging could not be acquired at follow-up. All patients were male, and the median age was 61 years (interquartile range (IQR) 54–67.5 years).

### Trial Oversight

The CheckRad-CD8 trial is registered at ClinicalTrials.gov (identifier: NCT03426657). The leading institutional review board at the Friedrich-Alexander-Universität Erlangen-Nürnberg (number: 131_18 Az) and all local ethic committees approved the CheckRad-CD8 trial. All patients gave written informed consent to all trial procedures, data protection measures, and the scientific use of imaging data.

### Definition of Pathological Complete Response

The results were correlated with either residual tumor or pathological complete response (pCR) after induction therapy before the initiation of radiation. pCR was defined as the complete absence of vital tumor cells in restaging biopsies. Biopsies were considered to represent the former tumor bed if significant regressive changes, i.e., fibrosis, bleedings, prominent chronic and active inflammation, were present.

### Image Analysis and Evaluation of Prognostic Relevance

In both baseline and follow-up multiparametric MRI, all lesions were manually segmented including the primary tumor (tumor volume) and lymph node metastases (lymph node volume) by a radiologist (KH, who was blinded to the clinical outcome) using Annotation Client (Chimaera GmbH, Erlangen, Germany) supervised by another radiologist with more than 15 years of experience in oncologic imaging (TB). The sequence used for delineation was the T1 weighted GRASP-VIBE sequence. The whole tumor volume was defined as the combined volume of the primary tumor and the lymph node metastases. The volume of the respective lesions was measured, and time-resolved mean intensities of the GRASP-VIBE sequence were extracted.

The GRASP-VIBE DCE values were analyzed with a custom-built R script. Using this script, the raw signal intensities of the first five DCE measurements were averaged and defined as a baseline with a relative enhancement set to 0. Bolus arrival was defined as the first measurement exceeding the raw signal intensity of the baseline by 5% and was set to 0 seconds. All measurements prior to the bolus arrival were omitted, and all subsequent measurements were normalized in terms of time (seconds since bolus arrival) and enhancement (relative to baseline enhancement).

The resulting data points were then fitted to a modified Brix equation (35):


$$\text{Enhancemen}{\text{t}}_{rel}=\text{A}*{\text{k}}_{ep}*\frac{{\text{e}}^{\left(-{\text{k}}_{ep}*\text{x}\right)}-{\text{e}}^{\left(-{\text{k}}_{\text{e}1}*\text{x}\right)}}{{\text{k}}_{\left\{\text{e}1\right\}}-{\text{k}}_{\left\{\text{ep}\right\}}},$$

where the relative enhancement and x (as the time in seconds) are known, while A, k_ep_, and k_el_ are to be determined. For this purpose, A, k_ep_, and k_el_ were iteratively approximated using the Levenberg–Marquardt least-squares minimization method until the algorithm converged at an optimal fit (36).

From the fitted curve, peak enhancement (PE) was determined as the maximum relative enhancement, with time to peak (TTP) defined as the corresponding x value in seconds. The maximum and minimum of the fitted curve’s first derivative were respectively defined as the wash-in and wash-out.

Further, the imaging features of T1 ratio (signal intensity of the lesion as compared to autochthonous back muscle intensity), STIR ratio (signal intensity of the lesion as compared to autochthonous back muscle intensity), and the ADC value were assessed using syngo.*via* (Siemens Healthineers) in a representative region of interest without cystic changes or cavitation. The response was evaluated using the pathohistological results of the week 4 biopsy samples. No residual tumor in the biopsy was considered as a complete response. DCE maps were created using the MR Tissue4D Analysis tool in syngo.*via*.

### Statistical Analysis

Statistical analyses were performed with IBM SPSS Statistics for Windows, version 24 (IBM Corp., Armonk, NY, USA). Nonparametric testing was performed with Kruskal-Wallis test and the Mann Whitney U test adjusted for multiple testing, if needed, for independent samples. Intrarater/retest reliability was measured using intraclass correlation coefficients (ICCs) for continuous measures. Five cases were randomly selected for reassessment by the rater. The interpretation of reliability results was based on the recommendations of Koo and Mae (37). Receiver operating characteristics (ROC), with respect to their area-under-the-curve (AUCROC), were compared using DeLong’s test. For all statistical tests, the level of significance was defined as p < 0.05. Confidence intervals were calculated at a confidence level of 95%.

### Predictive Modeling

The prediction of treatment response was regarded as a classification problem to be solved by a random forest algorithm, calculated in RStudio 3.4.1 (RStudio, Inc., Boston, MA, USA), using caret 6.0-81 (38). We chose a parallelized random forest as a classifier, as this algorithm is known to give stable and good results in different scenarios (39).

There were 15 parameters available as potential predictors for treatment response: A, k_ep_, and k_el_ from the Brix model; TTP, PE, area under the curve (AUC), wash-in, and wash-out (as parameters derived from the Brix equation); the T1 signal intensity (the raw measurement and the measurement normalized to muscle); the STIR signal intensity (raw measurement and normalized to muscle); ADC; p16-HPV-status; and patient age.

Feature selection was performed using a wrapper approach with a sequential backward selection based on parallel random forests (parRF). To account for class imbalances, the synthetic minority over-sampling technique was applied. The feature selection and training process was focused on maximizing AUCROC. The model was validated using a 10-fold cross-validation approach with 10 repeats.

The algorithm’s output is twofold, providing on the one hand probability values for the class assignment (range, 0-1), and on the other hand a dichotomous classification result (response *vs.* non-response). Hereby, the raw probability values were used to calculate the ROC curves, whereas the dichotomous classification resulted from applying a cutoff of 0.5 on the raw probability values. Sensitivity and specificity were then calculated based on the dichotomous classification results using a cutoff of 0.5. In principle, this cutoff could, however, be further adapted, e.g., to maximize the Youden-Index or to favor either sensitivity or specificity, depending on the clinical setting.

## Results

**Figure 1:**
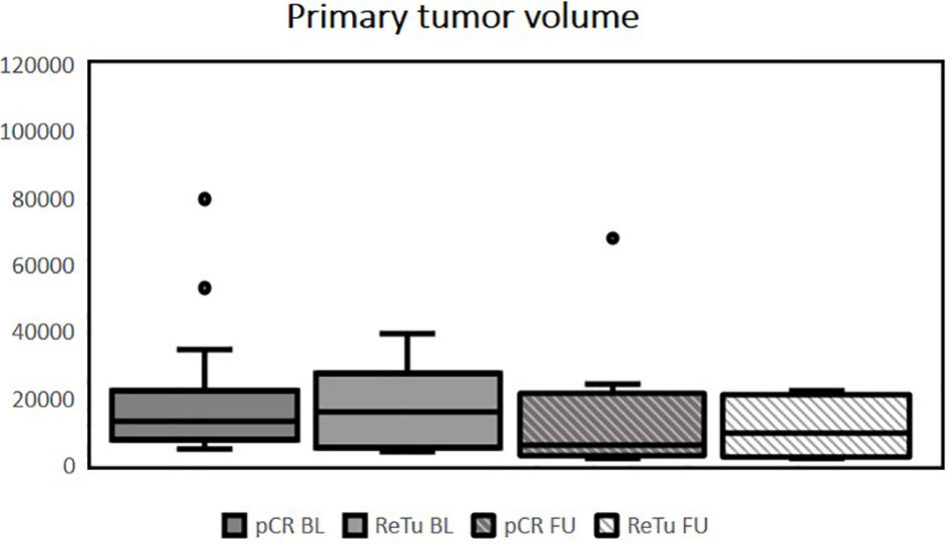
Tumor volume of the primary tumor in mm^3^, outliers are marked with dots, no significant differences among groups at baseline or follow-up were present using the Kruskal-Wallis test (pCR BL, pathologic complete response at baseline; ReTu BL, residual tumor at baseline; pCR FU, pathologic complete response at follow up; ReTu FU, residual tumor at follow up).

**Figure 2:**
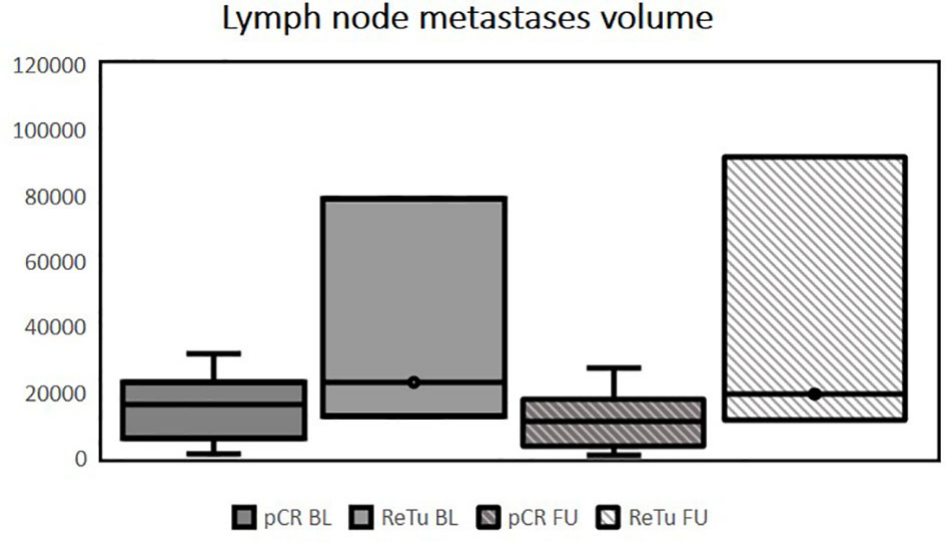
Tumor volume of the lymph node metastases in mm^3^, outliers are marked with dots, no significant differences among groups at baseline or follow-up were present using the Kruskal-Wallis_test (pCR BL, pathologic complete response at baseline; ReTu BL, residual tumor at baseline; pCR FU, pathologic complete response at follow up; ReTu FU, residual tumor at follow up). In the pCR group 9 patients showed lymph node metastases, in the ReTu group in 3 patient lymph node metastases were present.

**Figure 3:**
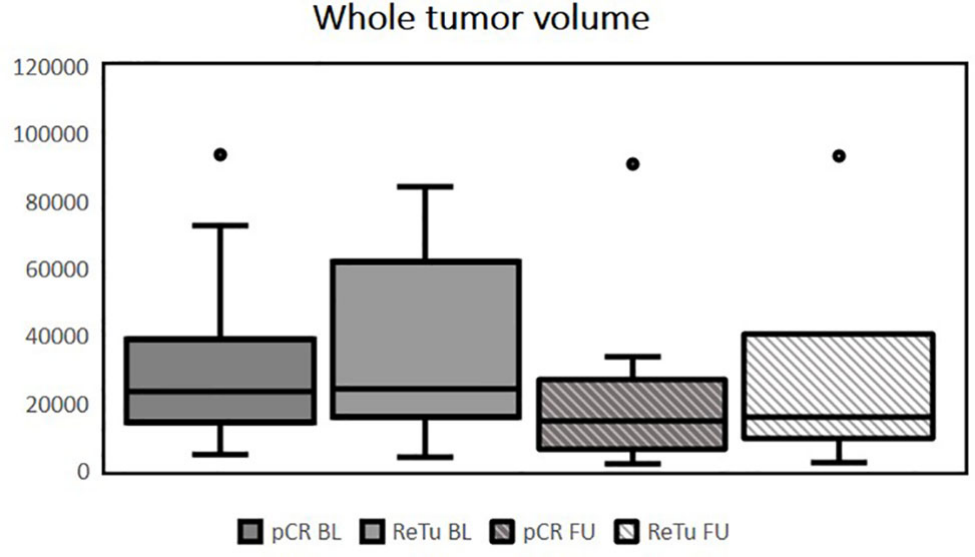
Whole tumor volume of both primary tumor and lymph node metastases in mm^3^, outliers are marked with dots, no significant differences among groups at baseline or follow-up were present using the Kruskal-Wallis test (pCR BL, pathologic complete response at baseline; ReTu BL, residual tumor at baseline, pCR FU, pathologic complete response at follow up; ReTu FU, residual tumor at follow up).

**Figure 4:**
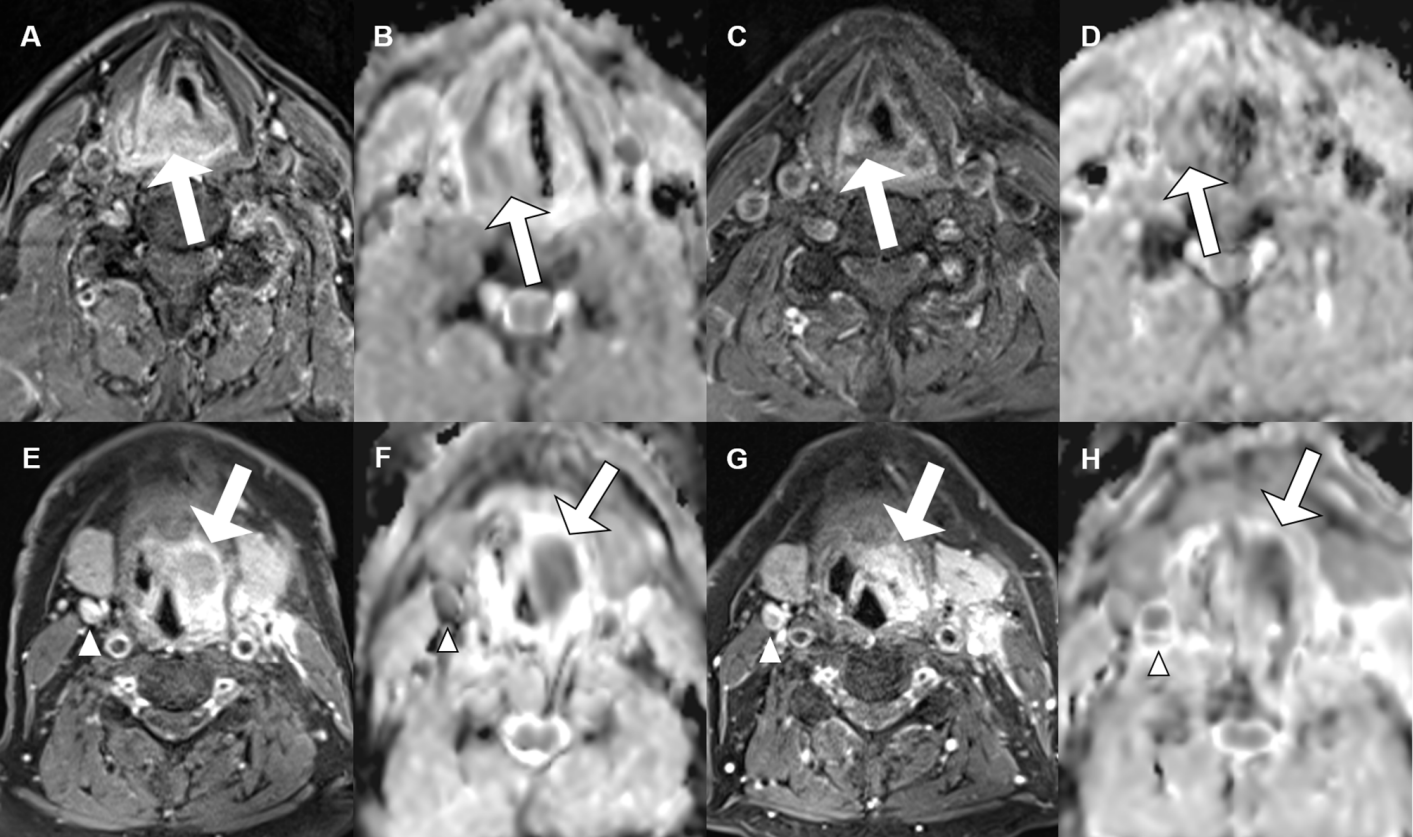
Morphological and functional MRI images for two patients with pharyngeal carcinoma (primary tumor is marked with an arrow, lymph node metastasis with an arrow head), contrast enhanced T1 weighted GRASP-VIBE sequence **(A, C, E, G)** and ADC map **(B, D, F, H)** for both a patient with pathologic complete response (upper row) and a patient with residual tumor (lower row) at baseline [**(A, B)** and respectively **(E, F)**] and at follow-up [**(C, D)** and respectively **(G, H)**].

**Figure 5:**
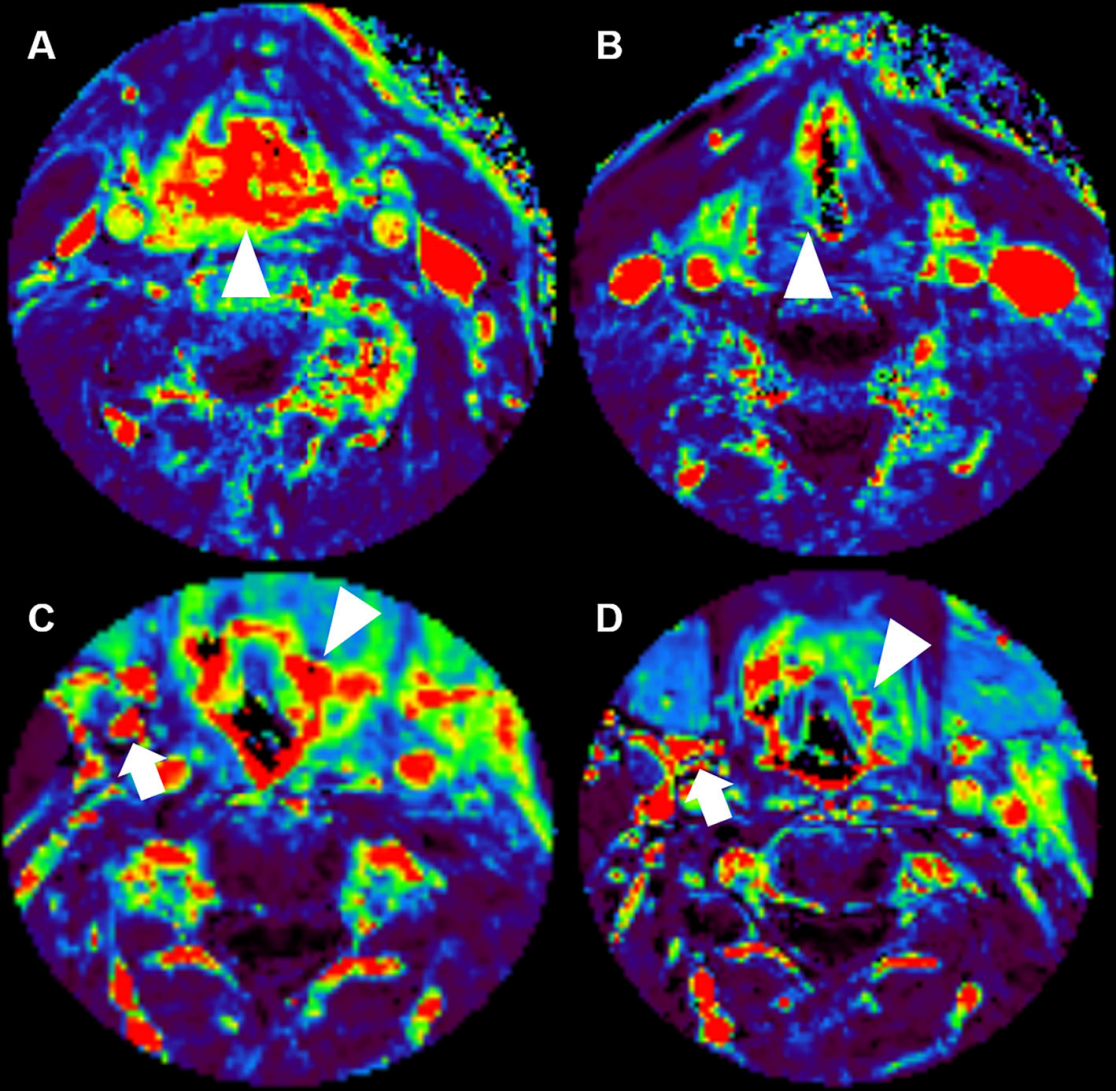
Corresponding positive enhancement integral maps of DCE MRI for the same patients as shown in , pathologic complete response (upper row) and residual tumor (lower row) at baseline **(A, C)** and at follow-up **(B, D)**. The area of the primary tumor is marked with an arrowhead, lymph node metastasis is marked with an arrow.

Patients had histologically confirmed HNSCC stage III–IVb of the oropharynx (n = 14), hypopharynx (n = 3), or supraglottic larynx (n = 3). Multilevel disease was present in two cases. Fifteen patients (68.2%) showed pCR in the re-biopsy. Of the seven patients with residual tumor (ReTu; 31.8%), six showed an inflamed immune phenotype and one biopsy showed an immune-excluded phenotype. Ten patients had human papillomavirus (HPV)-16-positive tumor tissue samples, there was no significant difference for HPV-16 positivity (p = 0.37). Lymph node metastases were present in 12 patients (54.5%). The median initial whole tumor volume (primary tumor plus lymph node metastases) before treatment was 23.7 cm^3^ (IQR 14.7–40.0). There was no significant difference among patients with pCR and patients with ReTu in whole tumor volume at baseline. The median whole tumor volume after therapy was 15.2 cm^3^ (IQR 6.4–28.2). The initial median volume of the primary tumor was 13.9 cm^3^ (IQR 7.2–23.5), and after treatment it was 6.0 cm^3^ (IQR 2.7–21.0). The initial whole tumor volume did not differ among pCR (median 23.4 cm³) and ReTu (median 24.0 cm³) patients at baseline (see **Table 1**). The median volume of the lymph node metastases at baseline and at follow-up, if present did not differ among groups (see **Table 1**). Primary tumor volume, whole tumor volume and lymph node metastases volume did not differ among groups at baseline and at follow-up (see **Figures 1**–**3**). Representative images of a patient with pCR and a patient of the ReTu group with lymph node metastasis show DCE MRI, morphological and diffusion weighted imaging illustrating the difficulty to determine responders by morphological criteria only (**Figures 4**, **5**).

**Table 1:** Response rate and tumor volume at baseline and follow up for all patients, pathologic complete response (pCR) group and residual tumor (ReTu) group.

	All patients	pCR	ReTu
Histological complete response	22	15	7
Median initial whole tumor volume (cm^3^, IQR 25 – 75)	23.7 (14.7 – 40.0)	23.4 (14.1 – 39.0)	24.0 (15.9 – 62.1)
Median whole tumor volume after therapy (cm^3^, IQR 25 – 75)	15.2 (6.4 – 28.2)	14.5 (6.1 – 26.9)	15.8 (9.5 40.4)
Lymph node metastases	12	9	3
Median initial lymph node metastases volume (cm^3^, IQR 25 – 75)	18.0 (9.5 – 25.4)	16.4 (5.7 – 23.0)	22.9 (12.5 – 79.0)
Median lymph node metastases volume after therapy (cm^3^, IQR 25 – 75)	11.5 (4.6 – 22.2)	11.1 (3.4 – 17.7)	19.5 (11.6 – 91.7)
Median initial primary tumor volume (cm^3^, IQR 25 – 75)	13.9 (7.2 – 23.5)	13.1 (7.2 – 22.3)	15.9 (5.2 – 27.2)
Median primary tumor volume after therapy (cm^3^, IQR 25 – 75)	6.0 (2.7 – 21.0) ^b^	5.8 (2.8 – 21.3)	9.5 (2.4 – 20.9)

No significant difference among patients with pathologic complete response (pCR) and patients with residual tumor (ReTu) at each timepoint (baseline or follow-up) was present, significance was defined as p < 0,05 with Kruskal-Wallis-Test corrected for multiple testing.

The correlation analysis of conventional MR imaging features at baseline (T1 ratio, STIR ratio, and ADC value) did not reveal any significant correlation with tumor response (see **Table 2**). Among pCR and ReTu patients, there was a significant difference in the STIR ratio measured in the primary tumor volume at follow-up (p = 0.007, see supplementary 1, raw values supplementary 2). For the pCR and ReTu group, there was no significant difference in the ADC value measured in the primary tumor volume and lymph node metastases at baseline or at follow-up (see supplementary 2). Intrareader agreement for volumetric measurements showed an ICC of 0.91 (95% CI 0.64–0.98), reflecting moderate to excellent agreement according to Koo et al. (37).

**Table 2:** Median and interquartile ranges (IQR) for DCE parameters at baseline, conventional MRI imaging features, ADC values in whole tumor volume and age with respective p-values using Wilcoxon signed rank-test and area under the curve of the Receiver Operating Characteristic (AUCROC).

	pCR	ReTu	p-value	AUCROC
**A**	1.967 (IQR 1.725–2.043)	2.126 (IQR 1.968–2.208)	0.2101	0.676
**kep**	0.046 (IQR 0.042–0.053)	0.048 (IQR 0.044–0.057)	0.7309	0.552
**kel**	-1.52×10-4 (IQR -2.8–3.9×10-4)	4.48×10-4 (IQR 1.8–4.9×10-4)	0.0659	0.752
**TTP**	276 (IQR 118–280)	102 (IQR 93–112)	0.04809	0.771
**PE**	2.02 (IQR 1.81–2.18)	2.04 (IQR 1.83–2.16)	0.8907	0.524
**AUC**	80.7 (IQR 68.8–85.1)	84.3 (IQR 80.6–89.0)	0.3322	0.638
**WIN**	0.087 (IQR 0.075–0.101)	0.096 (IQR 0.091–0.112)	0.2372	0.667
**WOUT**	3.06×10-4 (IQR -3.78–6.07×10-4)	-8.990×10-4 (IQR -9.42 – -3.68×10-4)	0.03194	0.79
**T1**	434 (IQR 396–512)	482 (IQR 461–490)	0.6298	0.571
**T1 Ratio**	1.11 (IQR 0.98–1.33)	1.04 (IQR 0.91–1.15)	0.4069	0.619
**STIR**	323 (IQR 273–377)	377 (IQR 277–437)	0.6216	0.571
**STIR Ratio**	4.90 (IQR 4.45–5.50)	4.21 (IQR 2.97–4.71)	0.1624	0.695
**ADC**	1029 (IQR 803–1372)	850 (IQR 822–1228)	0.7309	0.552
**Age**	60 (IQR 54–67)	61 (IQR 58–66)	0.6715	0.562

DCE parameters at baseline using the Brix model featured significant differences between the pCR and ReTu groups for TTP and wash-out (see **Table 2**). TTP and wash-out reached an AUCROC of 0.771 (95% CI 0.589–0.974) and 0.790 (95% CI 0.595–0.986), respectively.

**Figure 6:**
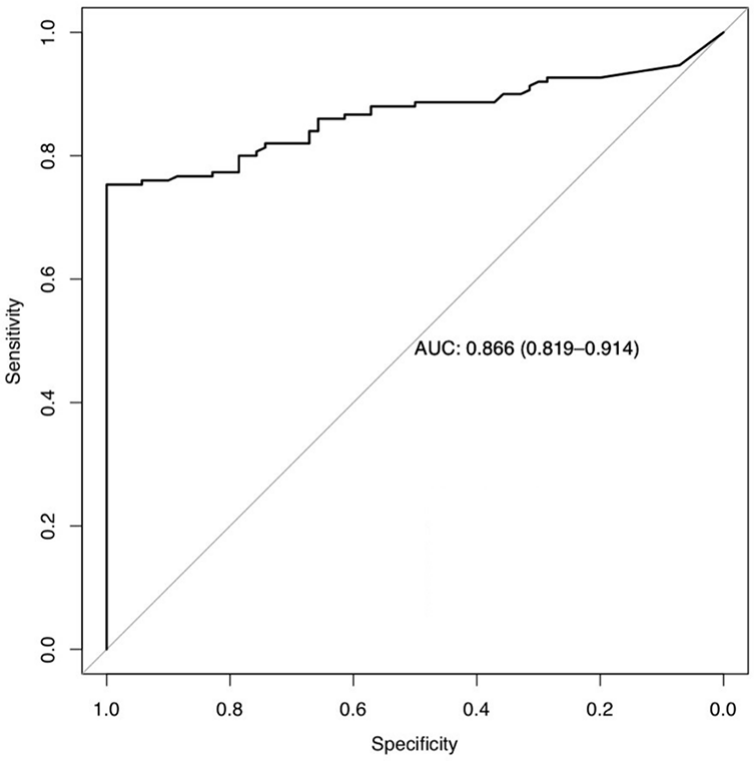
Area under the Receiver Operating Characteristic curve (AUC) and statistical data, in parentheses 95% confidence interval for the random forest model based on k_el_ und TTP.

Our model based on parRF, incorporating k_el_ and TTP from the Brix model, was able to predict therapy response with a sensitivity of 78.7% (95% CI 71.24–84.93) and a specificity of 78.6% (95% CI 67.13–87.48). The model had an AUCROC of 0.866 (95% CI 0.819–0.914) (see **Figure 6**). Although our model showed a higher AUCROC, it failed to significantly outperform its constituent parameters k_el_ and TTP.

## Discussion

In the current study, the primary tumor volume, whole tumor volume and volume of lymph node metastases did not differ between pCR and ReTu patients. Morphological criteria using the T1 and STIR ratios did not show a consistent association with therapy response, either, suggesting that conventional imaging parameters are not capable of evaluating immunotherapy treatment success after one follow-up. Additionally, the ADC value could not be used to assess response in our dataset. Therefore, the morphological and functional ADC parameters were not reliable response prediction criteria in our dataset.

Multiparametric MRI was used to create a predictive model of tumor response in HNSCC to ICI therapy at baseline using a random forest machine learning algorithm based on DCE parameters of the primary tumor in the baseline examination. The histopathological analysis four weeks after immunotherapy served as a standard of reference for therapy response. Due to tumor heterogeneity, VOIs instead of ROIs were employed. To our knowledge, this is the first study to employ the prognostic value of DCE MRI in head and neck tumors treated with induction chemo-immunotherapy. TTP and wash-out at baseline were significantly different between the pCR and ReTu group and could therefore be used to predict which patients would have a good response to ICI and chemotherapy treatment. The model based on parRF featured an even higher AUC (0.866) than the parameters alone, with a sensitivity of 78.7% and a specificity of 78.6%.

The introduction of immunotherapy has made the evaluation of tumor response increasingly difficult. In particular, pseudoprogression and mixed responses with both shrinkage and growth of primary tumors or metastases at follow-up are frequently observed, hampering the differentiation between remission and progression. The observation of 15 pCR in 22 treated patients reflects the possibility that induction chemo-immunotherapy may be more efficacious than classical induction chemotherapy (40).

In contrast to our results, the recent study of Borggreve et al. showed that ADC values and the SUV_mean_ in ^18^F-FDG PET/CT can help identify pathologic complete response to neoadjuvant chemotherapy in esophageal cancer (41). These different findings might be attributed to the different tumor entities and therapy regimens under investigation.

Possible solutions for assessing tumor response could be the use of invasive techniques, such as tumor biopsy, or incorporating more than one follow-up imaging study according to iRECIST criteria. Although a noninvasive method for immunotherapy response evaluation remains a challenge, it is crucial to develop cost-effective and personalized estimations of tumor response to ICI treatment. Several models using radiomics have been developed to evaluate tumor response; for example, a CD8 score using contrast-enhanced CT images was associated with tumor response in patients treated with ICIs and radiotherapy (42–44). Hao et al. applied multiparametric MRI in osteosarcoma patients at baseline and follow-up after neoadjuvant chemotherapy and surgery and was able to predict event-free survival and overall response using the DCE MRI parameter Ktrans (45). Our predictive model performed slightly better than those in other studies using radiomics in CT to predict response to ICIs. For example, Ligero et al. showed a sensitivity of 75% and a specificity of 53% in tumor types of various origins, such as breast, cervix, bladder, lung, and head and neck (46). Despite of the better performance our algorithm, the results did not differ significantly from the single DCE parameters used for the model.

Overall, our findings indicate that DCE parameters are promising for predicting immunotherapy treatment responses. Our results were histopathologically validated for every patient; nevertheless, further studies in larger populations should be performed. In a clinical context, the predictive value based on DCE MRI could facilitate the optimized selection of individual treatment options for each HNSCC patient using a noninvasive approach.

There are some limitations to this study: This was a single-center study with a limited number of patients enrolled. Because of this small sample size, the algorithm’s results should also be interpreted with caution. Ideally, one would have initially excluded a part of the data set as an independent test set, and trained the algorithm with the remaining data in order to validate its performance on the test set. However, with such a small number of samples, this approach was not an option. We thus decided to implement a particular cross-validation, which bears the risk of falsely estimating the algorithm’s performance too optimistically.

With these limitations in mind, the predictive model performed slightly better than the sole DCE parameters, however missing the significance threshold. For these reasons, a clinical application is limited at the moment. Performance measures with a higher degree of reliability will result from a larger sample size and the inclusion of an independent test set, both planned for follow-up studies.

Further limitations include the fact that the results are related to a fixed therapy regiment consisting of ICI and chemotherapy in HNSCC. The definition of pCR was based on a biopsy sample rather than the resected tumor. However, the samples were taken from regions that were metabolically active on FDG PET/CT before and after induction therapy.

In conclusion, we found that tumor volume, single morphological parameters, and the ADC values are of limited use for the evaluation of treatment response to chemo-immunotherapy in locally advanced HNSCC. However, a machine learning algorithm using parallel random forests based on DCE MRI parameters was able to predict treatment response following induction chemo-immunotherapy in HNSCC patients, but did not perform better than the DCE parameters alone.

## Data Availability Statement

The raw data supporting the conclusions of this article will be made available by the authors, without undue reservation.

## Ethics Statement

The studies involving human participants were reviewed and approved by leading institutional review board at the Friedrich-Alexander-Universität Erlangen-Nürnberg. The patients/participants provided their written informed consent to participate in this study.

## Author Contributions

KH, SE, MH, and TB designed the study. SR, SS, AG, BF, UG, and MH investigated the patients, had trial oversight and collected the samples. ME and AH conducted the histopathological examination. KH, SE, MW, and TB performed the image analysis and carried out the statistical analysis. HI, RF, AH, and MU supervised and substantially supported the acquisition of data based on their vast experience. KH, MH, and TB drafted the manuscript. All authors reviewed the manuscript critically and provided constructive comments to improve the quality of the manuscript. All authors read and approved the final manuscript.

## Funding

This work was supported and funded by AstraZeneca (ESR-16-12356). The trial was conducted as an investigator sponsored trial. The funding source did not influence the design, data collection, analysis, or interpretation. The manuscript was reviewed by the funding company. The corresponding author had full access to all the data and the responsibility for the decision to submit for publication. TB is supported by the German Research Foundation (Deutsche Forschungsgemeinschaft) within the Priority Programme µBone (BA 4027/10-1); by the Collaborative Research Centers 1181 Checkpoints for Resolution of Inflammation (CRC 1181, Project Z02); and by Transregio 305: Striking a moving target: From mechanisms of metastatic colonization to novel systemic therapies (CRC/TR 305, Project Z01).

## Conflict of Interest

MH reports the following conflicts of interest: Merck Serono (advisory role, speakers’ bureau, honoraria, travel expenses, research funding); MSD (advisory role, speakers’ bureau, travel expenses, research funding); AstraZeneca (research funding); Novartis (research funding); BMS (advisory role, honoraria, speakers’ bureau); and Teva (travel expenses). ME reports the following conflicts of interest: Diaceutics (employment, honoraria, advisory role, speakers’ bureau, travel expenses); Cepheid (research funding, advisory role); AstraZeneca (honoraria, advisory role, speakers’ bureau, travel expenses); Roche (honoraria, travel expenses); MSD (honoraria, speakers’ bureau); GenomicHealth (honoraria, advisory role, speakers bureau, travel expenses); Astellas (honoraria, speakers’ bureau); Janssen-Cilag (honoraria, advisory role, research funding, travel expenses); and Stratifyer (research funding, patents).). UG received support for presentation activities for Dr Sennewald Medizintechnik GmbH, has received support for investigator initiated clinical studies (IITs) from MSD and AstraZeneca and contributed at Advisory Boards Meetings of AstraZeneca and Bristol-Myers Squibb. SS reports the following conflicts of interest: stockholder of Siemens Healthineers.

The remaining authors declare that the research was conducted in the absence of any commercial or financial relationships that could be construed as a potential conflict of interest.

## Publisher’s Note

All claims expressed in this article are solely those of the authors and do not necessarily represent those of their affiliated organizations, or those of the publisher, the editors and the reviewers. Any product that may be evaluated in this article, or claim that may be made by its manufacturer, is not guaranteed or endorsed by the publisher.
