# Supplementary material for: Predictive Value of Multiparametric MRI for Response to Single-Cycle Induction Chemo-Immunotherapy in Locally Advanced Head and Neck Squamous Cell Carcinoma
Source: Front Oncol. 2021 Oct 21;11:734872. doi: 10.3389/fonc.2021.734872 (PMC8567752; doi:10.3389/fonc.2021.734872)
Supplement: Supplementary file 1 [file Table_1.docx]

|  | **Baseline** | **Follow up** |
| --- | --- | --- |
| **T1 ratio primary tumor** | 0.38 | 0.25 |
| **T1 ratio lymph node** | 0.52 | 0.52 |
| **STIR ratio primary tumor** | 0.15 | 0.007 |
| **STIR ratio lymph node** | 0.93 | 0.17 |
| **ADC value primary tumor** | 0.70 | 0.75 |
| **ADC value lymph node** | 0.12 | 0.78 |

supplementary 1 p-values for the comparison of T1 ratio, STIR ratio and ADC value between pathologic complete response group and residual tumor group at baseline and follow-up using Kruskal-Wallis test adjusted for multiple testing

|  | **Baseline** | **Follow-up** |
| --- | --- | --- |
| **T1 ratio primary tumor pCR** | 1.1 (IQR 0.95 – 1.3) | 1.2 (IQR 1.0 – 1.3) |
| **T1 ratio primary tumor ReTu** | 1.0 (IQR 0.87 - 1.2) | 1.1 (IQR 0.93 – 1.2) |
| **T1 ratio lymph node pCR** | 1.2 (IQR 0.98 – 1.4) | 1.3 (IQR 0.9 – 1.3) |
| **T1 ratio lymph node ReTu** | 1.2 (IQR 0.83 – 1.3) | 0.98 (IQR 0.95 – 1.2) |
| **STIR ratio primary tumor pCR** | 4.9 (IQR 4.4 – 5.5) | 3.5 (IQR 3.0 – 4.8) |
| **STIR ratio primary tumor ReTu** | 4.2 (IQR 2.8 – 4.9) | 2.9 (IQR 2.2 - 3.1) |
| **STIR ratio lymph node pCR** | 5.2 (IQR 4.5 -6.0) | 4.4 (IQR 3.5 – 5.9) |
| **STIR ratio lymph node ReTu** | 4.8 (IQR 4.8 – 9.1) | 5.8 (IQR 4.6 – 8.3) |
| **ADC value primary tumor pCR** | 1029 (IQR 795 – 1461) | 1292 (IQR 985 – 1602) |
| **ADC value primary tumor ReTu** | 850 (IQR 805 – 1297) | 1415 (IQR 998 – 1765) |
| **ADC value lymph node pCR** | 841 (IQR 686 – 864) | 1040 (IQR 836 – 1190) |
| **ADC value lymph node ReTu** | 944 (IQR 842 – 1100) | 1110 (IQR 825 – 1157) |

supplementary 2 median of T1 ratio, STIR ratio and ADC value between baseline and follow up separated for pathologic complete response (pCR) and residual tumor (ReTu) group (ADC value in 10^-6^ mm^2^/s)
